# Tympanomastoid cholesterol Granulomas presenting as a Blue Eardrum in children

**DOI:** 10.1186/s12893-023-02069-5

**Published:** 2023-06-13

**Authors:** Zhengcai Lou

**Affiliations:** Department of Otorhinolaryngology, Yiwu central Hospital, Yiwu city, 322000 Zhejiang provice China

**Keywords:** Cholesterol granuloma, Otitis media with effusion, Ventilation tube, Mastoidectomy, Eustachian tube dysfunction

## Abstract

**Objective(s):**

This clinical study was performed to analyze the characteristics of cholesterol granuloma (CG) and evaluate our results in children.

**Methods:**

The clinical records of children diagnosed with CG were retrospectively reviewed.

**Results:**

The total of 17 children (20 ears) with CGs were included in this study. Endoscopy revealed pars flaccida retractions and lipoid tissue deposition behind the intact blue tympanic membrane (TM). CT scan revealed bony erosion and extensive soft tissue in the middle ear and mastoid. No ossicular chain destruction was found. All 20 ears underwent canal wall-up mastoidectomy and ventilation tube (VT) insertion, 3 sets of VT were performed in 5 ear and 2 sets in one. The residual perforation was seen in 2 ears following VT. The CT revealed well-pneumatized antra and tympanic cavities at postoperative 12–24 months.

**Conclusion(s):**

The CG should be suspected for the patients with yellow lipoid deposition behind the blue TM. CT of CG usually revealed bony erosion and extensive soft tissue in the middle ear and mastoid. Mastoidectomy combined with VT insertion and etiological treatment have a favorable prognosis for CG in children.

## Introduction

Cholesterol granuloma (CG) of middle ear is rare in children; the first CG was reported by Manassé in 1894 [1]. The pathogenesis of the CG in children is unclear. A CG is usually considered to be related to chronic otitis media with effusion (OME) triggered by Eustachian tube (ET) dysfunction (ETD) [2–5]. OME, mucosal swelling, and hypertrophy create permanent granulation tissue that obstructs ear drainage [3]. The air in the mastoid process is absorbed, rendering the negative pressure of middle ear, in turn triggering ischemia, vascular rupture, and degradation of iron‑containing hemoglobin and other substances that can stimulate the foreign body response, that cannot be absorbed by giant cells [6]. CGs are intractable and irreversible, triggering a repetitive vicious cycle that further damages hearing and causes secondary cholesteatomas. The optimal CG treatment for children remains controversial. Some scholars have suggested staged ventilation tube (VT) insertion and mastoidectomy [6]; others recommended VT insertion combined with mastoidectomy and mastoid obliteration [7, 8]. This clinical study was performed to analyze the characteristics of cholesterol granulomas (CGs) and evaluate our results in children.

## Materials and methods

### Research ethics approval

The study was reviewed and approved by the Medical Research Ethical Committee of Yiwu central hospital. Informed consent was obtained from all participants’parents.

### Patients and methods

This study retrospectively examined 20 children with 24 blue tympanic membranes (TMs) and initially diagnosed with tympanomastoid CG which were treated surgically at our tertiary care referral center between January 2012 and January 2019. The inclusion criterias were the yellow lipoid tissue behind the intact blue TM, the history of OME with or without adenoidal or tonsillar hypertrophy; retraction of the pars flaccida and/or the pars tensa; preoperative computed tomography (CT) had evidence of soft tissue in the middle ear/mastoid; and a complete follow-up.

The exclusion criterias were age > 18 years; the history or evidence of TM perforation, otorrhea, trauma, or previous ear surgery; middle ear effusion; and histological evidence of a cholesteatoma.

All of included children had blue TM and CG as the main lesion, and all of them undergone concurrent adenotonsillectomy and mastoidecotomy combined with VT insertion under hospitalization.We analyzed demographic data, presenting symptoms, endoscopic examination, CT images, audiometric test data, surgical procedure, post operative course and outcomes.

The patients’s pre-operative hearing test included the pure tone audiometry, tympanography, and distortion product otoacoustic emission (DPOAE). The pure tone audiometry score was the average of the hearing thresholds at 0.5, 1, 2, and 3 kHz, and was calculated preoperatively and 12 months postoperatively. Postoperative followups were retrospectively evaluated, including endoscopic examination, complications, pure tone audiometry and CT at 12 months after surgery. CG was finally confirmed based on the intraoperative findings and histological examinations.

### Surgical procedure

All patients were placed under general anesthesia. Ablation or monopolar tonsillectomy and adenoidectomy were first performed on children in whom CGs were associated with adenoidal or tonsillar hypertrophy. Secondly, exploration of middle ear and the tympanum were performed that then led to an intraoperative decision to perform canal wall-up (CWU) or canal wall down (CWD) mastoidectomy. The antrum, aditus ad antrum, and/or mastoid were found to be filled with yellow lipoid tissue. This was removed but the normal mucosa was preserved. We did not involve the ossicular chain. The operated area was washed with saline, lipoid tissue suctioned away after tympanotomy, and a VT inserted. The EAC was not packed.

### Follow-up

Follow-up was scheduled for 2 and 4 weeks, and 3, 6, 9, 12, 18, and 24 months, after surgery. The TM, VT, and any effusion were examined endoscopically. Pure tone audiometry was performed 12 months after surgery. CT was examined following removal of VT or VT extrusion.The main outcomes were effusion, eardrum status, hearing improvement and VT expulsion. The secondary outcome was the soft tissue status (present/absent) of the middle ear/mastoid as revealed by CT.

## Results

### Initial diagnose

**Fig. 1:**
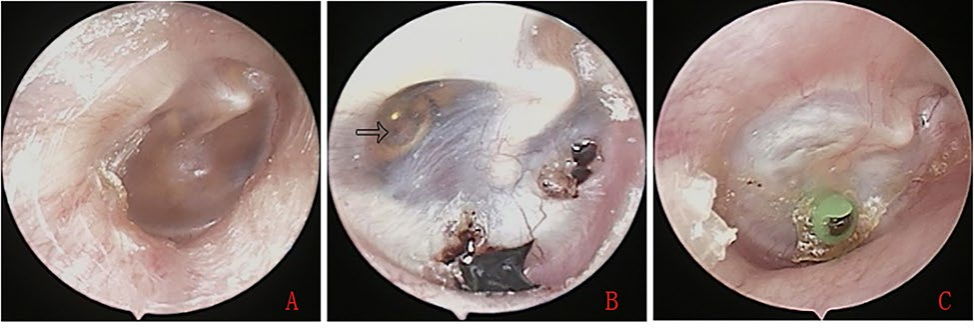
A-12 year-old boy. Endoscopic examination showed an right OME at first visit hospital, however, first treatment was refused (**A**), right OME with blue eardrums and lipoid tissue deposition of tympanic cavity (white arrows) at second visit hospital,time interval from the first visit was 9 months (**B**),and postoperative 7 months (**C**)

**Fig. 2:**
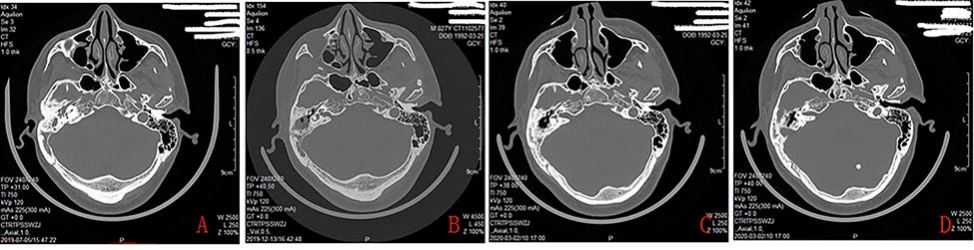
The preoperative CT revealled soft tissue density in the tympanic cavity, antrum, and aditus ad antrum (**A**), postoperative CT revealled well pneumatolytic middle ear (**B,C**, and **D**).Please note, this is the same patient as in Fig. 1

A total of 20 children with 24 ears (including unilateral ear in 16 children and bilateral ears in 4 patients) showed the blue TMs by endoscope and were initially suspected as CG. Of the 24 ears, 20 ears had yellow lipoid tissue behind the intact blue TM but 4 ears didn’t. All the 24 ears underwent the middle ear exploration. Although CT scan revealed extensive soft tissue without bony erosion in middle ear and mastoid in the 4 ears with no yellow lipoid tissue deposition, intraoperative exploration found no lipoid tissue deposition in the antrum, attic, mesotympanum, or mastoid. Thus, 3 patients with 4 ears were excluded in this study (Figs. 1 and 2), the total of 17 children with 20 ears (including unilateral ear in 14 children and bilateral ears in 3 patients) were finally diagnosed as the CG in this study.

### Demographic data

**Fig. 3:**
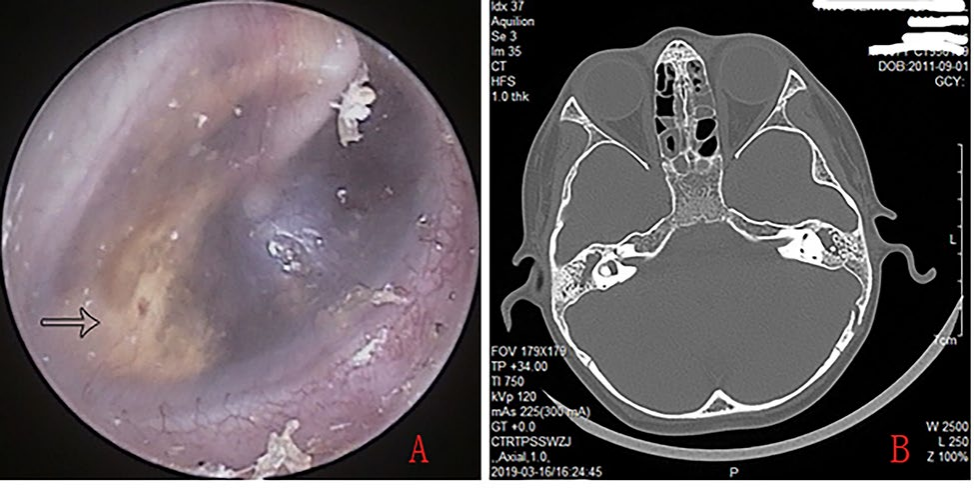
A-8 year-old boy. Endoscopic examination showed an right intact blue eardrums and yellow granulation tissue of tympanic cavity (white arrows) (**A**), CT revealed the bilateral soft tissue density in the antrum, aditus ad antrum and mastoid (**B**)

**Fig. 4:**
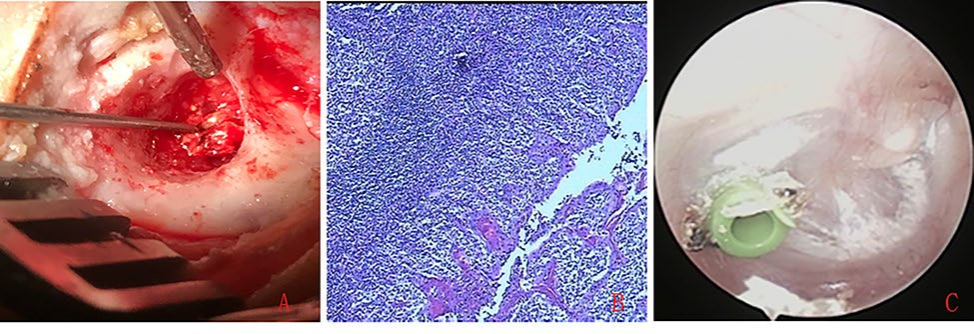
Intraoperative yellow cholesterol crystal (**A**), the cholesterol crystal with inflammatory granulation tissue was comfirmed by the histological examination (**B**), and postoperative 9 months (**C**).Please note, this is the same patient as in Fig. 3 The English in this document has been checked by at least two professional editors, both native speakers of English. For a certificate, please see: http://www.textcheck.com/certificate/vPTIxl

Of the 17 patients with 20 ears with CG, endoscopy revealed Type I attic retractions was 6 ears and type II in 14 ears as defined by Tos and Poulsen [8], pars flaccida retractions and yellow lipoid tissue deposition behind the intact blue TM (Fig. 3), the histological examination showed inflammatory granulation tissue and cholesterol crystals (Fig. 4).

Patients’ clinical and followup course showed in the Table 1. Of the 17 patients, 11 were in male and 6 in female, the average age was 8.23 ± 2.41 years (range 6–11 years). The left ear was affected in 8 patients, the right ear in 6, and both ears in 3. Of the 17 patients. 11 exhibited both adenoidal and tonsillar hypertrophy, 4 adenoidal hypertrophy, and 2 tonsillar hypertrophy. The concurrent adenotonsillectomy was performed on 11 patients, tonsillectomy in 2, and adenoidectomy in 4. No post-tonsillectomy hemorrhage was noted.

Of the 20 ears, hearing loss was the chief complaint in 20 ears, followed by tinnitus in 9 ears and otalgia in 3 ears. 19 had no history of paracentesis or VT insertion; 1 had previously received a VT that was extruded 3 months following surgery. All 20 ears failed DPOAE tests and demonstrated Type B tympanograms.

Of the 20 ears, CT scan revealed bony erosion and extensive soft tissue in the antrum, attic, and mesotympanum in 16 and in the antrum, attic, mesotympanum, and mastoid in 4 (Fig. 3). No ossicular chain destruction was found during the middle ear exploration. Subsequently, all 20 ears underwen CWP and VT insertion without involving the ossicular chain (Fig. 2).

**Table 1:** Patients’ clinical and followup course

Patient no	Side	Concurrent surgery		Mastoidectomy	VT extrusion		VT removal (post-op months)
					Onset (post-op months)	Treatment	
1	Right	adenotonsillectomy		CWU	/		13
2	Left	tonsillectomy	Allergic rhinitis	CWU	5	VTI + NS	
3	Left	adenotonsillectomy		CWU	/		12
4	Right	tonsillectomy		CWU	10	VTI	
5	Left	adenotonsillectomy	Allergic rhinitis	CWU	3	VTI + N	
6	Left	adenoidectomy		CWU	/		13
7	Left	adenotonsillectomy		CWU	/		13
	Right			CWU	/		14
8	Right	adenoidectomy		CWU	10	VTI	
9	Right	adenotonsillectomy		CWU	11	VTI	
10	Left	adenotonsillectomy		CWU	/		16
	Right			CWU	/		16
11	Right	adenoidectomy		CWU	/		13
12	Left	adenotonsillectomy	Allergic rhinitis	CWU	6	VTI + NS	
13	Left	adenotonsillectomy		CWU	/		14
14	Right	adenoidectomy		CWU	/		13
15	Left	adenotonsillectomy		CWU	11	VTI	
	Right			CWU	9	VTI	
16	Left	adenotonsillectomy	Allergic rhinitis	CWU	8	VTI + NS	
17	Left	adenotonsillectomy		CWU	12	VTI	

No: number; VTI:ventilation tube insertion; post-op: post-operative; CWU:canal wall up; NS: nasal spray

### Postoperative outcomes

All 17 patients with 20 ears were followed-up for 12–24 months, which exhibited mild otorrhoea for 1–3 weeks after surgery; this ceased by 1 month for 7 ears, 2–3 months for 11 ears, and 4 months for 2 ears. Of the 17 patients, only 14 completed pre-and post-operative pure tone audiometry (3 were uncooperative); the average air-bone gap was 27.3 ± 5.1 dB before surgery and 10.8 ± 4.7 dB after surgery.

Of the 20 ears, VT extrusion occurred in 10 ears during followup; while VT extrusion didn’t occur but VTs were removed from the remaining10 ears between 12 and 16 months after surgery, CT revealed well-pneumatized antra and tympanic cavities. The success rate without need for a repeated VT insertion was 50%(Table 1).

Of the 10 ears with VT extrusion, CT revealed soft tissue in the middle ear, the extrusion occurred 3–8 months after surgery from 4 ears with allergic rhinitis, recurrent OME developed, the exudate was removed after tympanotomy, and VTs reinserted, intranasal steroids and montelukast were then prescribed for 12 months, and no otorrhoea developed; the VTs were removed. However, the extrusion occurred 9–12 months from the remaining 6 ears, 3 sets of VT were performed in 5 ear and 2 sets in one.

No facial nerve palsy or intratympanic VT was observed during follow-up. The residual perforation was seen in 2 ears following removal of VT.

## Discussion

Most scholars believe that CG pathogenesis in children likely reflects an ETD that causes middle ear ventilation to fail [2–5]. It is difficult to differentiate CG from OME in children.Yang et al. [9]believed that CG was a late complication of repeated myringotomy or grommet insertion of chronic OME. However, 19 had no history of paracentesis or VT insertion; only 1 had previously received a VT in this study. Thus, our finding was similar to Miura et al’s study that CG is a sequela of chronic OME [5].

In this study, 24 ears exhibited blue TMs, while only 20 ears had lipoid tissue deposition and were histologically diagnosed as the CG, in addition, the blue TMs vanished after CG and effusion clearance, and VT insertion. The mechanisms of blue TM is unclear. Some authors have sought to explain the blue TM using the “exposed-marrow” theory, the hypoxia, negative pressure and inflammation of the middle ear resulted in the exposed marrow bleeds and coagulates and obstruction within the mucosal lining, thereby caused a secondary cyst [10–12]. However, our endoscopic examation and surgical exploration found that all the lipoid tissue was deposited in the middle ear cavity not the the mucosal lining. We speculated that the blue TM could be secondary to the bleeds or thrombosis of capillary in the middle ear mucosa. The persistent obstruction of ET creates severe negative pressure in aditus, antrum, and middle ear, followed by mucosa edema, capillary bleeding of mucosa in the middle ear and blood clot deposition in the middle ear cavity.This mechanism is similar to the sinus mucosal hemorrhage associated with aerosinusitis and the middle ear mucosa bleeding observed during hyperbaric oxygen therapy [13–16]. The mucosal bleeds depends on the degree of the ET obstruction and pressure change. In addition, the persistent bleeds and accumulation of blood clot would result in the thrombosis and slow resorption of lipid, thereby cause CG. Some scholars believed that the CG formation is closely related to the thrombosis [17–19]. Thus, blue TM is not specific manifestation of CG but the early stage, while the presence of blue TM affects prognosis or outcome of OME. However, CG should be warned for the children with yellow lipoid tissue deposition behind the blue TM. We speculated that CG formation includes the following stages: persistent obstruction of ET, OME, blue TM, lipoid tissue deposition, and CG formation. The exact mechanisms of CG formation remains to be further studied.

Although all TMs exhibited pars flaccida and pars tensa retractions,curiously, no adhesions were noted during intraoperative exploration. Maeta et al.[7] suggested that both CG and adhesive OM were caused by ETD, but TM changes were different. CT scan is useful to help differentiate CG from OME. Although CT scan revealed extensive soft tissue in the middle ear and mastoid in the CG and OME, we found that CG presented the bony erosion but OME didn’t. Usually, MRI as a diagnostic tool is more superior than CT in diagnosis of cholesterol granuloma, however, unfortunately, MRI wasn’t performed in this study.

No consensus CG treatment has yet emerged. Some authors believe that VT insertion alone improves CG [6], while others suggested mastoidectomy combined with VT insertion [7, 20–22]. In Matsuda et al’s study [6],one patient who underwent VT insertion alone expelled the VT 3 months later; the CG was unchanged. Ou YK, et al. [23] compared the surgical effect of CWU alone, CWU combined with VT and CWU combined with Balloon dilation eustachian tuboplasty (BDET), the success rate was12.5%, 52.6%, and 63.6% respectively. In present study, of the 20 ears with CG underwent adenotonsillectomy combined with CWU, exploration of the tympanum, and VT insertion, the VT extrusion occurred in 10 ears which require multiple sets of VT; while VTs were removed from the other 10 ears between 12 and 16 months after surgery. This results suggested that a 50% success rate of this technique without need for a repeated VT insertion.

Kudoh et al. [24] believed that a CG developed in response to a middle ear blockage. As the granulation tissue became hyperplastic or engaged in hypersecretion, the middle ear pressure became positive, triggering persistent otorrhoea and VT expulsion. Even if otorrhoea ceased after VT insertion alone in few patients, it is possible that a cholesterol-rich effusion could progress to a CG. Thus, some scholars concluded that treatment of CG should feature (at least) thorough mastoidectomy and VT insertion [7]. The Japanese consensus is that mastoidectomy or VT insertion alone is inadequate; both procedures are required [22]. VT insertion not only compensates well for ETD but also drains residual postoperative effusion following CPU. Adenoidal and tonsillar hypertrophy are the principal causes of ETD and OME in children. The simultaneous adenotonsillectomy was performed to allows the ET to recover in this study. However, VT extrusion occurred 3–8 months after surgery from 4 ears with allergic rhinitis, 9–12 months from 6 ears, CT revealed soft tissue in the middle ears. VTs reinsertion and simultaneous intranasal steroids were applied for 12 months, the VTs were then removed for the 4 ears with allergic rhinitis. Thus, it is essential to eliminate the inducing factor of ETD for the treatment of CG. In addition, some scholars suggested that complete mastoidectomy is sometimes impossible [7, 20], granulation tissue regrowth from remaining cells or postoperative effusion may trigger hypersecretion and fluid collection, in turn expelling the VT. The present study suggested that patients who expel VTs soon after surgery require CT to explore the status of the middle ear. Repeat VT expulsion or obstruction can easily occur if CT reveals a middle ear shadow after VT reinsertion, that should be endoscopically removed and a VT reinserted.

The ossicular erosion is uncommon despite the extensive middle ear pathology in CG patients. Some authors reported that only 5/16 and 2/66 patients had the ossicular erosion and required reconstruction of the ossicular chain [6, 25]. In this study, no ossicular chain destruction was found. This results were similar to other studies [7, 23]. Maeta M, et al. [7] reported no ossicular erosion in 11 patients with CG, Ou YK, et al. [23] also did not find the ossicular erosion in 49 patients with CG. In present study, the ossicular chain is not affected by the pathology in all cases, we believed that the erosion degree of the ossicular chain is related to the disease course of CG, all the patients with CG were diagnosed in the early stage. However, the exact mechanism is not very clear and needs more investigations.

The limitations of the study was small size of sample and no control group. In addition, although MRI is more accurate than CT in the diagnosis of CG and its diffrentiation from cholesteatoma and other diseases, MRI was not performed in this study, which resulted in application of the middle ear exploration in 3 patients with 4 blue TMs.

## Conclusions

The CG should be suspected for the patients with yellow lipoid deposition behind the blue TM. CT of CG usually revealed bony erosion and extensive soft tissue in the middle ear and mastoid. Mastoidectomy combined with VT insertion and etiological treatment have a favorable prognosis for CG in children.

## Data Availability

All data generated or analyzed during this study are included in the published article.
